# Fluorouracil-induced leukoencephalopathy mimicking neuroleptic malignant syndrome: a case report

**DOI:** 10.1186/s13256-023-03814-3

**Published:** 2023-03-08

**Authors:** Pasin Hemachudha, Wanakorn Rattanawong, Thanakit Pongpitakmetha, Warongporn Phuenpathom

**Affiliations:** 1 Thai Red Cross Emerging Infectious Diseases Health Science Centre, World Health Organization Collaborating Centre for Research and Training on Viral Zoonoses, King Chulalongkorn Memorial Hospital, Faculty of Medicine, Chulalongkorn University, Bangkok, 10330 Thailand; 2 Division of Neurology, Department of Medicine, King Chulalongkorn Memorial Hospital, Faculty of Medicine, Chulalongkorn University, Bangkok, 10330 Thailand; 3 Department of Medicine, Faculty of Medicine, King Mongkut’s Institute of Technology Ladkrabang, Bangkok, Thailand; 4 Department of Pharmacology, Faculty of Medicine, Chulalongkorn University, Bangkok, 10330 Thailand; 5 Chula Neuroscience Center, King Chulalongkorn Memorial Hospital, Faculty of Medicine, Chulalongkorn University, Bangkok, 10330 Thailand; 6 Chulalongkorn Center of Excellence for Parkinson’s Disease and Related Disorders, Chulalongkorn University Hospital, Bangkok, 10330 Thailand

**Keywords:** 5-FU, Leukoencephalopathy, Neurotoxicity, Chemotherapy, Neuroleptic malignant syndrome

## Abstract

**Background:**

Fluorouracil-induced leukoencephalopathy is a rare complication and has been reported to present as confusion, oculomotor abnormality, ataxia, and parkinsonism; however, there is no previous report of a presentation mimicking neuroleptic malignant syndrome. Acute cerebellar syndrome may occur, which can be explained by the extremely high accumulation of the drug in the cerebellum. However, presentation mimicking neuroleptic malignant syndrome similar to our case has never been reported.

**Case presentation:**

Here, we describe a 68-year-old Thai male presenting with advanced-stage cecal adenocarcinoma, as well as symptoms and signs indicative of neuroleptic malignant syndrome. He received two doses of intravenous metoclopramide 10 mg 6 hours before his symptoms occurred. Magnetic resonance imaging scan revealed signal hyperintensity within the bilateral white matter. Further evaluation showed that his thiamine level was extremely low. Thus, he was diagnosed with fluorouracil-induced leukoencephalopathy mimicking neuroleptic malignant syndrome. The concomitant fluorouracil-induced thiamine deficiency eventually leads to rapid depletion of thiamine and was considered a risk factor for fluorouracil-induced leukoencephalopathy.

**Conclusion:**

Fluorouracil-induced leukoencephalopathy is believed to be caused by insult causing mitochondrial dysfunction. However, the exact mechanism remains unknown, but our finding suggests that thiamine deficiency plays a crucial role in fluorouracil-induced leukoencephalopathy. Diagnosis is usually delayed due to a lack of clinical suspicion and results in significant morbidity requiring unnecessary investigations.

**Supplementary Information:**

The online version contains supplementary material available at 10.1186/s13256-023-03814-3.

## Background

Fluorouracil (5-FU) is the main component of regimens for head and neck, breast, pancreatic, and colorectal carcinomas. Neurotoxicity caused by 5-FU rarely occurs but is a well-known side effect of the medication [1]. However, acute cerebellar ataxia does occur and is explained by the extremely high accumulation of the drug in the cerebellum [2, 3]. Other rarer side effects include oculomotor abnormality, optic neuropathy, stroke-like presentation, and movement disorders [4–8]. 5-FU-induced neurotoxicity can also cause leukoencephalopathy, and diagnostic features include encephalopathy that develops during or after the administration of medication. However, presentation mimicking neuroleptic malignant syndrome (NMS) similar to our case has never been reported.

NMS is a neurologic emergency associated with dopamine-receptor blocking agents, most often occurring with first-generation antipsychotics. It is characterized by a change in the level of consciousness, rigidity, fever, and dysautonomia. The onset of NMS is as early as 1 day after the initiation or dose increment of medication but can occur years into therapy, and its symptoms gradually evolve over 24–72 hours [9]. Its diagnosis is based on the Levenson criteria, incorporating physical signs and laboratory tests with three major criteria [fever, rigidity, and elevated creatine phosphokinase (CPK)] and five minor criteria (tachycardia, abnormal blood pressure, altered consciousness, diaphoresis, and leukocytosis) [10]. The diagnosis requires the presence of three major, or two major and four minor criteria.

## Case presentation

**Fig. 1:**
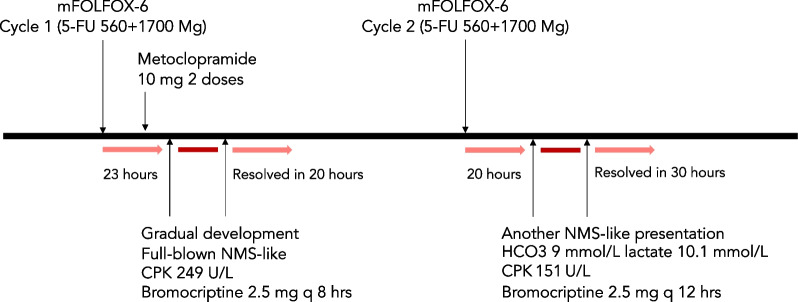
Timeline of patient’s medication administration, symptoms, and disease progression. Each cycle was performed 2 weeks apart, producing identical symptoms

A 68-year-old Thai male presented with advanced-stage cecal adenocarcinoma accompanied by liver and peritoneal metastases. He also exhibited symptoms of rigidity, tremor, and dysphagia, which progressed to tachypnea, tachycardia, diaphoresis, and eventually confusion over 20 hours. One day before his symptoms occurred, he was admitted for the first cycle of chemotherapy with a standard dose of modified FOLFOX-6 (mFOLFOX-6: oxaliplatin, 5-fluorouracil, and leucovorin), 400 mg/m^2^ of 5-FU administered as bolus, and 1200 mg/m^2^ infusion in 23 hours on days 1 and 2. His symptoms started approximately 1 day after the bolus dose administration and peaked during day 2 of infusion. He received two doses of intravenous metoclopramide 10 mg 6 hours before his symptoms occurred (Fig. 1). No other medication was given, and the patient was previously fully independent and did not exhibit neurologic abnormality.

The patient’s vital signs were as follows: body temperature 36.9 °C, blood pressure 169/113 mmHg, regular heart rate 110 beats per minute, respiratory rate 24 breaths per minute, and oxygen saturation 100% on room air. Physical examination revealed mild cachexia, diaphoresis, and confusion. Systemic examination results were otherwise within normal limits. Neurologic examination showed marked rigidity at the bilateral arms and legs (Additional file 1: Video S1). No tremor (including the perioral area), myoclonus, or dystonia was observed. The patient was confused but was able to follow simple one-step commands. Spastic dysarthria was also observed. The results of other cranial nerve examinations were normal. Both pupils were 3 mm equal and reactive to light bilaterally. The patient’s resting eye position was in the midline with normal spontaneous eye movement in all directions. The result of fundoscopic examination was normal with no papilledema. The rest of the neurologic examination results were normal, including the deep tendon reflexes. The patient was diagnosed with NMS as a result of generalized rigidity and acute confusional state. The initial blood test revealed mildly elevated CPK level of 249 mg/dL, mild anemia, hemoglobin level of 11.2 g/dL, leukocytosis, total white blood cell count of 15.5 × 10^9^/L (neutrophil, 84%), and acidosis with bicarbonate of 17 mEq/L. Metoclopramide rarely causes NMS, especially when it develops acutely within less than a day. However, given the findings of leukocytosis, and because no other explanation can be provided, he was administered oral bromocriptine 2.5 mg every 8 hours and intravenous crystalloids.

The patient regained his consciousness after 6 hours, and his motor activity returned to normal after less than a day. He was discharged with the diagnosis of improved mild NMS. Oral bromocriptine was tapered to every 12 hours after 1 week. However, further episodes of the described event in this patient occurred 2 weeks apart after receiving the second cycle of mFOLFOX-6 (Fig. 1). Metoclopramide or anti-psychotic drugs were not given at that time, which prompted reconsideration of the diagnosis. This identical episode occurred less than 1 day after receiving the same chemotherapy regimen (Additional file 2: Video S2).

**Fig. 2:**
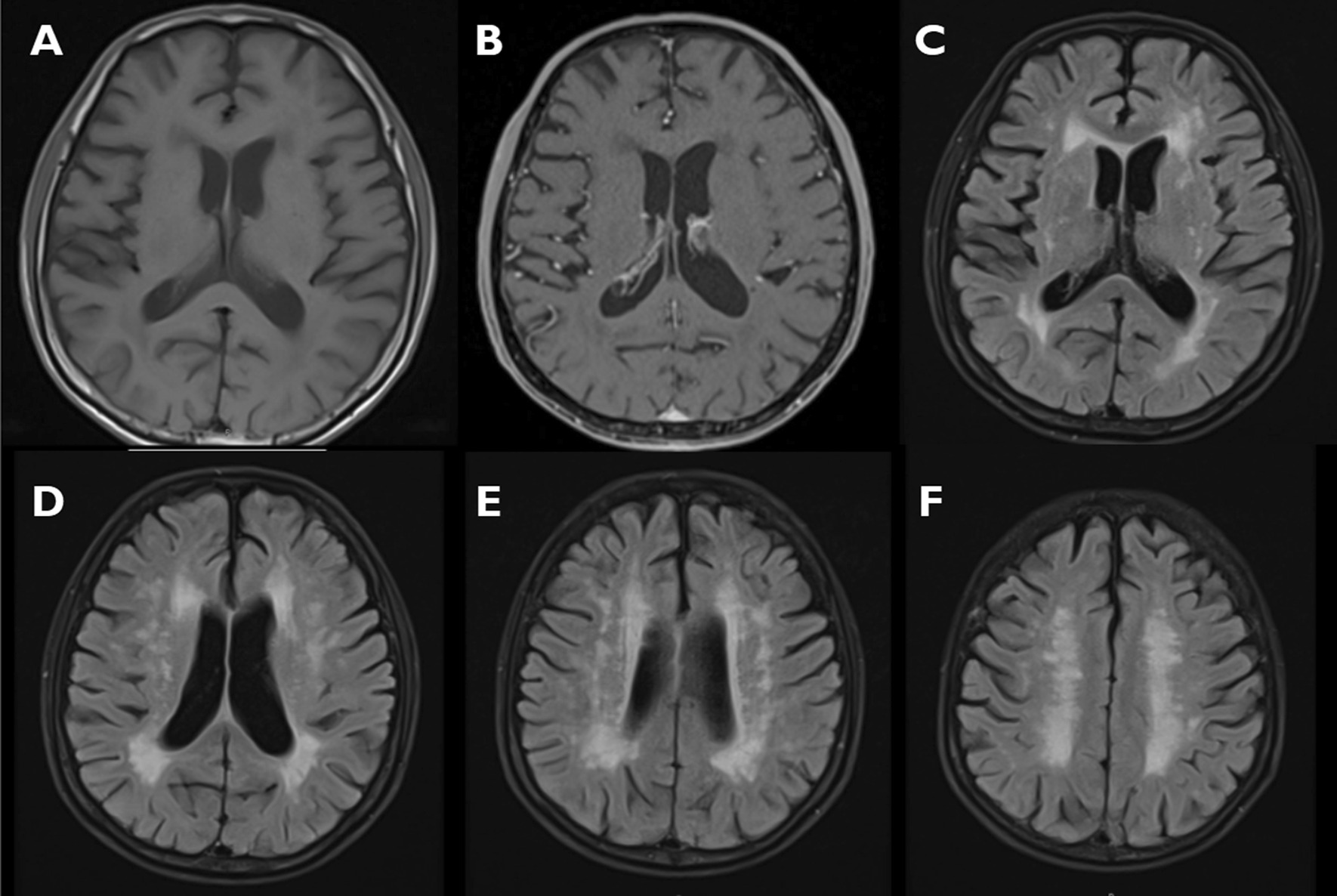
MRI brain T1-weighted sequence (**A**), T1-weighted sequence with gadolinium (**B**), T2-fluid-attenuated inversion recovery (**C**–**F**). MRI was performed 2 days after the second course of mFOLFOX6. The study on T2 fluid-attenuated inversion recovery demonstrated multiple nonspecific periventricular and deep subcortical white matter changes in the bilateral cerebral hemispheres, as well as internal and external capsules. Mild cerebral volume loss is noted. No space-taking lesion, restricted diffusion, or abnormal enhancement on gadolinium study

His blood test revealed CPK level of 151 mg/dL, marked leukocytosis, total white blood cell count of 17.1 × 10^9^/L (neutrophil, 87%), lactic acidosis with bicarbonate of 9 mEq/L, and arterial lactate of 10.1 mEq/L. Chest radiograph did not show infiltration, and his urinalysis result was completely normal. His blood culture later was also negative. He was resuscitated given his abnormal blood tests, and bromocriptine was restarted with an improvement of his symptoms within 2 days and total resolution after 3 days. Nevertheless, the patient’s CPK level and the recurrent event prompted reconsideration of the diagnosis. An MRI scan of the brain revealed widespread white matter change consistent with leukoencephalopathy (Fig. 2). The levels of serum thiamine (thiamine pyrophosphate) and ammonia were 10.9 μg/dL (28–85 μg/dL) and 32 μg/dL (30–120 μg/dL), respectively. Dihydropyrimidine dehydrogenase (DPD) testing was not performed as it is not readily available in our hospital. The thiamine level was found to be very low; thus, supplementation was initiated with intravenous thiamine 200 mg once daily for 3 days, and he was continued on oral thiamine 100 mg per day. Lumbar puncture was not conducted as the symptoms rapidly resolved. The diagnosis of 5-FU-induced acute leukoencephalopathy was established, and the patient’s third cycle of 5-FU was adjusted to 600 mg/m^2^ continuous infusion on days 1 and 2, which is half the original dose of 5-FU, and concomitant high-dose intravenous thiamine 500 mg every 8 hours for 3 days with no issue.

## Discussion

5-FU, a fluorinated analog of uracil, is one of the most commonly used cytotoxic chemotherapies and is the main component of regimens for head and neck, breast, pancreatic, and colorectal carcinomas. It acts mainly as a thymidylate synthase inhibitor, blocking the conversion of deoxyuridine monophosphate to deoxythymidine monophosphate required for DNA replication, resulting in cellular arrest and cell death [11]. An additional mechanism is the direct misincorporation of a 5-FU metabolite, fluorouridine triphosphate, into the tumor cell RNA, causing RNA damage and cell apoptosis [11].

Neurotoxicity is a rare (0.9–7%) but well-known side effect of 5-FU as the medication can easily cross the blood–brain barrier [1]. An acute cerebellar syndrome with ataxia, dysmetria, dysarthria, and nystagmus occurs in older regimens involving consecutive administration of 5-FU. It is explained by the extremely high accumulation of the drug in the cerebellum [2, 3]. Other rarer side effects are linked to DPD deficiency. DPD is a rate-limiting enzyme in the catabolism of 5-FU, and deficiency of this enzyme results in a reduced drug elimination process and toxic level accumulation. The side effects include oculomotor abnormalities with impaired convergence and divergence, optic neuropathy, stroke-like presentation, and movement disorders, such as parkinsonism and focal dystonia [4–8]. However, presentation mimicking NMS, similar to our case, has never been reported.

5-FU-induced neurotoxicity can cause leukoencephalopathy. *In vitro* and *in vivo* studies demonstrated that short-term systemic 5-FU damaged the lineage-restricted progenitor cells of the central nervous system (CNS) and nondividing oligodendrocytes, leading to acute CNS toxicity. 5-FU could alter transcriptional regulation in the oligodendrocytes and cause extensive myelin damage, which leads to delayed demyelinating cerebral complication [12]. Diagnostic features include encephalopathy developed during or after medication administration and exclusion of other metabolic causes, such as infection and hepatic or renal disorders [13]. Symptoms usually start at a median of 19.5 hours (range 10–30 hours) after infusion, and complete recovery can be observed within 15 hours of stopping the medication (range 3–72 hours) [13, 14]. DPD deficiency may be one of the predilections for the development of leukoencephalopathy. However, two hypotheses have been currently proposed to explain the process. The first one is that the rapid increase in the level of ammonia from a by-product of 5-FU leads to tricarboxylic acid (TCA) cycle suppression, resulting in urea cycle impairment, and thus direct neuronal toxicity [15]. The TCA cycle suppression also explains the finding of hyperlactatemia in our patient. However, increased ammonia level alone is unlikely to explain the degree of white matter changes. Therefore, we hypothesized the concomitant of the second theory, which is 5-FU-induced thiamine deficiency. The mechanism of the event involved 5-FU-induced systemic cellular dysfunction impairing DNA processing, thereby inducing a hypermetabolic or defective state with mitochondrial impairment and suppressed TCA cycle [16, 17]. 5-FU could increase thiamine pyrophosphate, which is an active metabolite of thiamine [17]. Therefore, these events eventually lead to rapid thiamine depletion from the cellular compensation mechanism [17]. In our case, thiamine depletion was considered a risk factor for 5-FU-induced leukoencephalopathy, given the low thiamine level and the alleviation of further neurotoxicity with concomitant thiamine administration. Several case reports have found thiamine levels similar to our study, thus rendering thiamine deficiency more likely [18, 19]. However, more studies are warranted to prove the exact pathogenesis of 5-FU-induced leukoencephalopathy. The risk for 5-FU-related leukoencephalopathy is probably multifactorial and ranges from dehydration, preexisting liver disease that predisposes to hyperammonemia, and superimposed infection and malnutrition that further predispose to thiamine deficiency [13, 14].

In our patient, the presentation mimicking NMS is very atypical for 5-FU-induced leukoencephalopathy and can be explained by two hypotheses. First, 5-FU may cause neurotoxicity to the dopaminergic neurons of the nigrostriatal, mesolimbic, and mesocortical pathways, resulting in presentation mimicking NMS [20]. However, the damage is not so severe as to create lesion on the basal ganglion in MRI of the brain. Second, because the patient was in an acute confusional state, it was difficult to differentiate between spasticity and rigidity. While this patient may have generalized spasticity from his leukoencephalopathy, this hypothesis is less likely due to normal reflexes and absence of long-tract signs.

Management of 5-FU CNS toxicities involves discontinuation of 5-FU administration, prompt supportive treatment, and monitoring of possible complications in the next chemotherapy cycle. Prompt and appropriate management usually lead to rapid symptom resolution; however, delayed detection might lead to high morbidity and mortality rates [21]. Uridine triacetate (Vistogard), an oral antidote for 5-FU approved by the US Food and Drug Administration, mainly competes with the toxic 5-FU metabolite for incorporation into RNA in the normal tissues; thus, it could be given as soon as possible (usually within 96 hours) to reduce severe 5-FU toxicity and overdose [22]. However, uridine triacetate is not widely available worldwide, including in our country.

## Conclusion

The diagnosis of 5-FU-induced leukoencephalopathy is often delayed due to heterogeneity in symptom presentation and exclusion of possible etiologies. The presentation mimicking NMS, along with low CPK levels, in a patient who received 5-FU prompts the recognition of 5-FU-induced leukoencephalopathy. Several mechanisms, such as TCA cycle suppression, DPD deficiency, hyperammonemia, and thiamine deficiency, probably play a role in neurotoxicity. The recognition of atypical signs and symptoms are important for general physicians and movement disorder specialists to avoid unnecessary investigation and provide appropriate care in a timely manner.

## Supplementary Information

**Additional file 1: Video S1.** Neurologic examination showed confusion with marked rigidity at the bilateral arms and legs.

**Additional file 2: Video S2.** The recurrent episode of confusion with marked rigidity at the bilateral arms and legs occurred 20 h after receiving the second cycle of mFOLFOX-6. Metoclopramide or anti-psychotic drugs were not given at that time.

## Data Availability

Not applicable.
